# Clinical and Radiologic Characteristics of Human Metapneumovirus Infections in Adults, South Korea

**DOI:** 10.3201/eid2501.181131

**Published:** 2019-01

**Authors:** Hyun Jung Koo, Han Na Lee, Sang Ho Choi, Heungsup Sung, Hwa Jung Kim, Kyung-Hyun Do

**Affiliations:** University of Ulsan College of Medicine, Seoul, South Korea (H.J. Koo, S.H. Choi, H. Sung, H.J. Kim, K.-H. Do); Kyung Hee University Hospital, Seoul (H.N. Lee)

**Keywords:** human metapneumovirus, viruses, pneumonia, respiratory infections, computed tomography, transplantation, outcome, infections, clinical characteristics, radiologic characteristics, radiography, South Korea

## Abstract

Clinical features of human metapneumovirus (HMPV) infection have not been well documented for adults. We investigated clinical and radiologic features of HMPV infection in 849 adults in a tertiary hospital in South Korea. We classified patients into groups on the basis of underlying diseases: immunocompetent patients, solid tumor patients, solid organ transplantation recipients, hematopoietic stem cell transplant recipients, hematologic malignancy patients, and patients receiving long-term steroid treatment. Of 849 HMPV-infected patients, 756 had community-acquired infections, 579 had pneumonia, and 203 had infections with other pathogens. Mortality rates were highest in hematopoietic stem cell transplantation recipients (22% at 30 days). Older age, current smoking, and underlying disease were associated with HMPV pneumonia. Body mass index and an immunocompromised state were associated with 30-day mortality rates in HMPV-infected patients. Bronchial wall thickening, ground-glass opacity, and ill-defined centrilobular nodules were common computed tomography findings for HMPV pneumonia. Macronodules and consolidation were observed in <50% of patients.

Human metapneumovirus (HMPV), first described in 2001, is a common pathogen that causes acute respiratory tract infections in all age groups (*1*). Seropositivity for IgG against HMPV has been detected in up to 100% of persons 20 and >65 years of age, and reinfection is common (*2*–*4*). HMPV infection shows a seasonal pattern in the United States, Asia, and countries in Europe, and most infections occur in spring (*3*,*5*–*9*). Although HMPV infection is usually asymptomatic or causes mild and self-limiting symptoms in young healthy adults, it can cause severe pneumonia in elderly and immunocompromised persons (*10*–*13*). HMPV infection progresses from upper respiratory tract infection (URI) to lower respiratory tract disease in up to 60% of hematopoietic stem cell transplant (HCT) recipients (*14*), and mortality rates are 6%–40% (*15*,*16*). Moreover, ≈50% of patients with solid organ transplants (SOT) infected with HMPV progress to pneumonia (*13*,*17*,*18*), and HMPV infection is frequently detected in patients with exacerbated chronic obstructive pulmonary disease (*19*).

Clinical characteristics such as host immunity in patients with HMPV infection and radiologic findings of HMPV pneumonia are needed for early detection of HMPV infection and for studies of HMPV pneumonia-related outcomes (*14*,*20*,*21*). Although a recent study of 3 long-term care facilities in Japan reported clinical and radiologic characteristics of HMPV pneumonia, that study did not assess the proportion of URIs, included only immunocompetent persons, and did not determine overall outcomes of HMPV pneumonia. Therefore, we conducted a study that included a large consecutive cohort of adults infected with HMPV and assessed the proportions of HMPV-associated URI and pneumonia in patients with various underlying disease, and laboratory findings, radiologic findings, including computed tomography (CT) images, and overall outcomes.

## Methods

This retrospective consecutive cohort study covered the period January 2010–February 2016. The study was approved by the institutional review board of Asan Medical Center (approval no. 2017–0016), which waived the requirement for informed consent because of the retrospective nature of this study. During the study period, all patients who came to this hospital, regardless of whether they were in the outpatient clinic, hospitalized, or in the emergency department, and who had respiratory symptoms suggestive of URI or pneumonia underwent routine collection of nasopharyngeal swab specimens, blood cultures, or both. Testing decisions were made by the clinicians. We assessed pathogens before giving any antimicrobial drugs to patients with no history of treatment at another hospital. If relevant pathogens could not be identified, bronchoalveolar lavage (BAL) fluid was obtained. BAL fluid was not obtained if there was no evidence of pneumonia and symptoms were eliminated by conservative management. The decisions for laboratory testing and BAL procedures were clinician directed, and laboratory results were assessed retrospectively.

During January 2010–February 2016, a total of 15,311 patients had tests performed for respiratory virus infections. For these patients, 817 patients had multiple tests because of multiple different episodes (> a 2-month interval between tests); 591 patients had 2 tests, 149 had 3 tests, 46 had 4 tests, 15 had 5 tests, 10 had 6 tests, 3 had 7 tests, 1 had 8 tests, and 2 had 9 tests. The total number of tests was 16,489. If a patient was infected more than once with HMPV during the study period, we used only the first episode for analysis. We thoroughly reviewed electronic medical records of patients, and their clinical characteristics; immune status, such as transplant history and steroid or immunosuppressant use; presence of other pathogens; length of hospital stay; and clinical course, such as admission to an intensive care unit; and death.

### Definitions

Community-acquired infection indicated respiratory infection detected in a person in a community without a history of hospitalization or living in a long-term care facility within the previous 14 days. Hospital-acquired infection was defined as respiratory infection that occurred >48 hours after hospital admission with new onset respiratory symptoms. We assessed respiratory virus infection in nasopharyngeal secretions and BAL fluid by using a multiplex reverse transcription PCR and a Seeplex RV 15 ACE Detection Kit (March 2010–October 2013) or an Anyplex II RV 16 Detection Kit (November 2013–August 2017) (both from Seegene Inc., http://www.seegene.com).

Patients positive for HMPV, but with no evidence of pneumonia by chest radiographs or CT images, were defined as having URI. Patients who had new pulmonary infiltrates by chest radiographs or CT images and HMPV in nasopharyngeal samples or BAL fluid were defined as having pneumonia. Patients with other pathogens in nasopharyngeal or blood samples within 2 days of diagnosis of HMPV infection were defined as co-detection positive for another pathogen (*14*). Long-term steroid use was defined as steroid treatment (>5 mg/d of prednisolone or an equivalent drug) for >6 months because of an underlying condition or disease (Appendix Table 1, https://wwwnc.cdc.gov/EID/article/25/1/18-1131-App1.pdf), such as adrenal insufficiency, interstitial lung disease, or asthma. The mean dose of steroid was 1.80 mg/kg/d (range 0.53–2.02 mg/kg/d).

### Radiologic Evaluation

For patients given a diagnosis of HMPV infection, we evaluated the presence of pneumonic infiltrates on chest radiographs to detect pneumonia. We also evaluated bilaterality and the number of involved zones (total of 6 zones; i.e., right and left upper, middle, and lower zones).

We performed CT examinations by using 16- or 64-detector CT scanners (SOMATOM Sensation 16; Siemens Medical Solutions, https://www.siemens.com/global/de/home.html; and LightSpeed VCT; General Electric Healthcare, https://www.gehealthcare.com). We reviewed all axial and coronal CT images on the picture archiving and communication system by using the mediastinal (width, 450 hounsfield units [HUs]; level, 50 HUs), lung (width, 1,500 HUs; level, −700 HUs), and bone (width, 1,000 HUs; level, 200 HUs) window settings.

We assessed CT findings for distribution of parenchymal abnormalities (number of involved lobes and bilaterality); the presence and extent of centrilobular nodules; consolidation; ground-glass opacities; and the presence of macronodules, bronchial wall thickening, bronchiectasis, lymphadenopathy, and pleural effusion. CT patterns were defined on the basis of the glossary of terms for thoracic imaging (*22*). All CT results were reviewed in consensus by 2 chest radiologists (1 with 2 years of experience and 1 with 15 years of experience) in thoracic imaging. Results were independently reviewed by a third radiologist to evaluate the reliability of the CT findings (percent extent of centrilobular nodules, consolidation, and ground-glass opacities).

### Statistical Analysis

Patients were subgrouped according to underlying conditions: immunocompetent patients, patients with solid tumors, SOT recipients, HCT recipients, patients with hematologic malignancy (HM), and patients receiving long-term steroid treatment. We compared characteristics and outcomes of HMPV infection for each of these groups. We compared proportions of HMPV pneumonia in immunocompetent and immunocompromised patients by using the χ^2^ test. Univariate and multivariable logistic regression analyses were performed to identify clinical factors associated with HMPV pneumonia and the 30-day mortality rate in HMPV-infected patients. Body mass index (BMI) was analyzed as a continuous variable. We assessed interobserver agreement for CT findings by determining intraclass correlation coefficients with κ statistics. We compared categorical variables by using the χ^2^ test or Fisher exact test. We compared continuous variables by using the Student *t*-test or the Mann-Whitney U test. CT findings for HMPV pneumonia without another pathogen were compared in immunocompetent and immunocompromised patients. A 2-sided p value <0.05 was defined as statistically significant. We performed all statistical analyses by using SPSS version 21.0 (SPSS Inc., https://www.ibm.com/analytics/spss-statistics-software).

## Results

### Patient Characteristics

**Figure 1:**
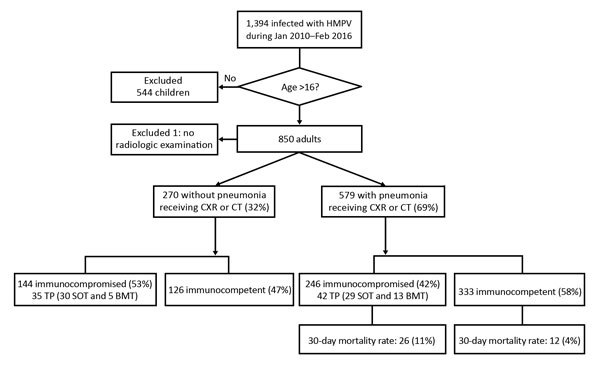
Flowchart for analysis of clinical and radiologic characteristics of adults with HMPV infections, South Korea. BMT, bone marrow transplant; CT, computed tomography; CXR, chest radiograph; HMPV, human metapneumovirus; SOT, solid organ transplants; TP, transplant.

**Figure 2:**
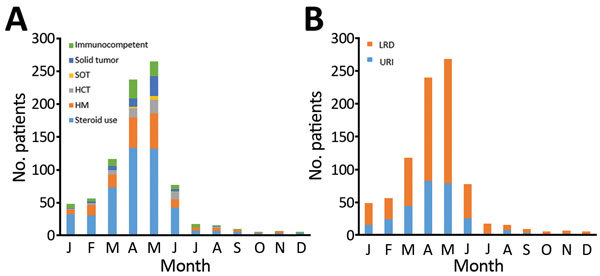
Analysis of clinical and radiologic characteristics of adults with human HMPV infections by monthly distribution of HMPV infection, South Korea. A) Rates of underlying diseases; B) proportions of upper respiratory tract infection and lower respiratory tract disease. HCT, hematopoietic stem cell transplantation; HM, hematologic malignancy; HMPV, human metapneumovirus; LRD, lower respiratory tract disease; SOT, solid organ transplants; URI, upper respiratory tract infection.

For the study period, January 2010–February 2016, we identified 850 adults infected with HMPV (Figure 1). There was 1 patient in the long-term steroid use group who had 2 episodes, and the interval between the 2 episodes was 1 year. The overall percentage of patients with HMPV infection among all those tested was 5.6% (850/15,311). HMPV was detected in 5.2% (851/16,489) of all tests. Most (82.0%, 696/850) patients were given a diagnosis during March–June (Figure 2). One patient who did not undergo radiologic examination was excluded, and 579 (68.2%) of the 849 HMPV-infected patients were given a diagnosis of pneumonia. For pneumonia patients, 14 patients with negative results for chest radiographs had pneumonia on the next CT scan. The percentage of pneumonia in immunocompetent patients (72.5%, 333/459) was slightly higher than that for immunocompromised patients (63.1%, 246/390) (p = 0.003).

We determined characteristics of the HMPV-infected patients (Table 1). Of the 849 patients, 459 were immunocompetent, 174 had solid tumors, 59 had a history of SOT, 9 underwent HCT, 58 had underlying HM, and 90 had a history of long-term steroid use. We provide details of their underlying diseases (Appendix Table 1). Median age of immunocompetent HMPV-infected patients was 67 years, which was higher than that for the solid tumor, SOT, HCT, and HM patient groups. Most (89%) HMPV infections were community-acquired; the remaining infections (11%) were nosocomial. C-reactive protein concentration (6.5 mg/dL vs. 2.6 mg/dL; p<0.001), leukocyte count (7.5 × 10^3^ cells/μL vs. 6.5 × 10^3^ cells/μL; p = 0.001), and neutrophil count (5.5 × 10^3^ cells/μL vs. 4.3 × 10^3^ cells/μL; p<0.001) were significantly higher in patients with pneumonia than in those without pneumonia.

**Table 1:** Clinical characteristics of patients infected with human metapneumovirus, South Korea*

Characteristic	Immunocompetent	Solid tumors†	SOT	HCT	HM	Steroid use	Total
Total	459 (54)	174 (20)	59 (7)	9 (1)	58 (7)	90 (11)	849
Age, y	67 (56–75)	62 (53–70)	56 (49–62)	54 (32–63)	60 (47–67)	67 (52–75)	64 (53–73)
Sex							
M	229 (50)	89 (51)	25 (42)	6 (67)	24 (41)	50 (56)	442 (52)
F	230 (50)	85 (49)	34 (58)	3 (33)	34 (59)	40 (44)	407 (48)
Smoking status							
Never	326 (71)	104 (60)	43 (73)	5 (56)	40 (69)	47 (52)	565 (67)
Former	99 (22)	63 (36)	16 (27)	3 (33)	14 (24)	38 (42)	233 (6)
Current	34 (7)	7 (4)	0	1 (11)	4 (7)	5 (6)	51 (27)
BP, mm Hg							
Systolic	134 (120–152)	124 (115–137)	131 (116–146)	130 (113–136)	128 (120–140)	134 (120–151)	131 (119–147)
Diastolic	80 (73–90)	80 (73–88)	82 (74–92)	88 (82–90)	82 (76–90)	82 (74–90)	80 (73–90)
Temperature, °C	36.6 (36.4–37.2)	36.6 (36.4–37.2)	36.4 (36.2–36.8)	36.7 (36.5–37.7)	36.5 (36.2–36.7)	36.6 (36.3–36.9)	36.6 (36.4–37.1)
Temperature, >37°C	70 (15)	25 (14)	4 (7)	3 (33)	5 (9)	16 (18)	123 (14.5)
Heart rate, beats/min	83 (70–99)	88 (78–103)	82 (73–91)	100 (90–110)	87 (77–100)	93 (84–106)	87 (74–100)
Respiratory rate, breaths/min	20 (18–22)	20 (18–20)	18 (18–20)	22 (20–27)	20 (18–22)	22 (20–24)	20 (18–22)
Body mass index, kg/m^2^	23.2 (20.4–25.9)	23.1 (20.3–25.5)	23.2 (21.3–25.1)	20.2 (17.0–25.1)	22.4 (20.5–25.2)	22.6 (20.2–24.4)	23.1 (20.4–25.5)
Hypertension	239 (52)	71 (41)	47 (80)	3 (33)	22 (38)	51 (57)	433 (51)
Diabetes mellitus	157 (34)	42 (24)	44 (75)	4 (44)	20 (35)	29 (32)	296 (35)
Location of patients‡							
Outpatient clinic	22	1	2	0	0	2	27
Hospitalized	131	137	21	3	24	35	351
ED	306	36	36	6	34	53	471
Type of infection							
CA	415 (90)	150 (86)	51 (86)	7 (88)	47 (81)	84 (93)	756 (89)
Nosocomial	44 (10)	24 (14)	8 (14)	2 (22)	11 (19)	6 (7)	93 (11)
URI	126 (28)	68 (39)	30 (51)	1 (11)	20 (35)	27 (30)	270 (32)
LRD	333 (73)	106 (61)	29 (49)	8 (89)	38 (66)	63 (70)	579 (68)
Initial CRP level at hospital admission, mg/L	4.3 (1.5–9.9)	6.5 (2.6–13.3)	4.6 (1.0–7.7)	10.6 (5.5–19.7)	5.8 (2.8–12.0)	4.4 (1.9–4.4)	4.9 (1.8–11.0)
Leukocyte count at diagnosis, × 10^3^/μL	7.7 (5.5–10.5)	5.4 (2.8–8.7)	8.5 (4.8–10.6)	12.0 (6.5–13.6)	4.8 (2.3–7.4)	8.3 (5.7–11.5)	7.2 (4.9–10.3)
>10	128 (28)	33 (19)	16 (27)	5 (56)	7 (12)	34 (38)	223 (26)
<10	331 (72)	141 (81)	43 (73)	4 (44)	51 (88)	56 (62)	626 (74)
Neutrophil count at diagnosis, × 10^3^/μL§	5.5 (3.4–8.3)	3.9 (1.7–6.5)	6.1 (3.3–8.5)	7.9 (5.9–9.5)	2.9 (1.3–5.1)	6.2 (3.9–9.6)	5.1 (3.0–8.1)
>5.0	252 (55)	67 (39)	35 (59)	6 (67)	14 (24)	56 (62)	430 (51)
<5.0	205 (45)	106 (61)	24 (41)	1 (11)	42 (72)	32 (36)	410 (48)
Lymphocyte count at diagnosis, × 10^3^/μL§	1.3 (0.9–1.8)	0.8 (0.5–1.3)	1.0 (0.6–1.4)	2.9 (0.7–4.2)	0.8 (0.4–1.7)	1.1 (0.7–1.6)	1.1 (0.7–1.6)
>0.7	380 (83)	97 (56)	41 (70)	5 (56)	33 (57)	65 (72)	621 (73)
<0.7	77 (17)	76 (44)	18 (31)	2 (22)	23 (40)	23 (26)	219 (26)
Platelet count, × 10^3^/μL	193 (145–243)	149 (101–223)	166 (116–231)	82 (35–187)	86 (36–146)	198 (143–245)	176 (124.5–233)
Blood urea nitrogen, mg/Dl	14 (10–22)	13 (9–19)	21 (14–31)	15.0 (8.5–27.0)	14.5 (10–20.3)	15 (11–23.5)	14.0 (10.0–21.0)
Creatinine, mg/dL	0.8 (0.7–1.1)	0.8 (0.6–1.0)	1.3 (1.0–1.8)	0.9 (0.7–1.4)	0.8 (0.6–1.2)	0.8 (0.6–1.1)	0.8 (0.7–1.1)
Procalcitonin, ng/mL	0.2 (0.1–1.2)	0.2 (0.1–0.7)	0.2 (0.1–1.2)	0.2 (0.1–1.1)	0.2 (0.1–0.6)	0.2 (0.1–0.9)	0.2 (0.1–0.9)

*Values are no. (%) or median (interquartile range) unless otherwise indicated. BP, blood pressure; CA, community acquired; CRP, C-reactive protein; ED, emergency department; HCT, hematologic stem cell transplantation; HM, hematologic malignancy; HMPV, human metapneumovirus; LRD, lower respiratory tract disease; SOT, solid organ transplants; URI, upper respiratory tract infection.  †Patients with solid tumors were given chemotherapy within 6 mo. ‡Locations at which patients were tested for HMPV. §Detailed laboratory results, including neutrophil and lymphocyte counts, were unavailable for 9 patients who visited outpatient clinics

Overall, 23.9% (203/849) of patients had a pathogen other than HMPV, and 16.5% (140/849) had pneumonia and another pathogen (Appendix Table 2). Rates for other pathogens ranged from 20% to 33% in subgroups and were highest for patients with HM (32.8%, 19/58) and those receiving long-term steroid treatment (32.2%, 29/90). Bacteria were the most common co-detected pathogens (57.1%, 116/203).

Of the HMPV-infected patients, 65% were hospitalized for a median of 7 days (range 4 days–13 days) (Table 2). An antiviral agent (oral ribavirin) was used in 129 patients, and intravenous immunoglobulin was used in 11 patients. For patients hospitalized for pneumonia, 68 (8%) required admission to the intensive care unit. HCT recipients had the highest all-cause 30-day (22%) and 90-day (33%) mortality rates.

**Table 2:** Co-detection of other pathogens and clinical course of patients infected with human metapneumovirus, South Korea*

Characteristic	Immunocompetent	Solid tumors	SOT	HCT	HM	Steroid use	Total
Total	459 (54)	174 (20)	59 (7)	9 (1)	58 (7)	90 (11)	849
URI	126 (28)	68 (39)	30 (51)	1 (11)	20 (35)	27 (30)	270 (32)
LRD	333 (73)	106 (61)	29 (49)	8 (89)	38 (66)	63 (70)	579 (68)
Ribavirin use	44 (10)	22 (13)	10 (17)	5 (56)	23 (40)	22 (24)	126 (15)
IVIG use	3 (1)	0	1 (2)	0	1 (2)	3 (3)	8 (1)
Both ribavirin and IVIG use	2 (0.4)	0	0	0	0	1 (1)	3 (0.4)
Co-detection of other pathogen, n = 126							
URI	17 (13)	18 (26)	10 (33)	1 (100)	10 (50)	7 (26)	63 (23)
LRD	72 (21)	23 (22)	13 (45)	1 (13)	9 (24)	22 (35)	140 (24)
Bacteria	48	20	16	1	11	20	116
Virus	32	14	7	0	6	5	64
Fungi	3	5	0	1	0	0	9
Bacteria and virus	6	1	0	0	2	3	12
Bacteria and fungi	0	1	0	0	0	0	1
Virus and fungi	0	0	0	0	0	1	1
Hospital admission	279 (65)	98 (56)	32 (54)	6 (67)	35 (60)	64 (71)	552 (65)
Length of hospital stay, days	6.0 (4.0–13.0)	7.0 (4.0–12.0)	6.0 (4.0–14.0)	7.0 (5.3–55.3)	10.5 (5.8–32.5)	7.0 (4.0–16.0)	7.0 (4.0–13.0)
ICU admission	42 (9)	4 (2)	5 (10)	1 (11)	5 (10)	11 (12)	68 (8)
All-cause mortality rate at 30 d	16 (3)	17 (10)	0 (0)	2 (22)	5 (9)	7 (8)	47 (6)
All-cause mortality rate at 90 d	18 (4)	22 (13)	1 (2)	3 (33)	7 (12)	8 (9)	59 (7)
Overall mortality rate	42 (9)	34 (20)	3 (5)	4 (44)	21 (36)	22 (24)	126 (15)

*Values are no. (%) or median (interquartile range) unless otherwise indicated. HCT, hematologic stem cell transplantation; HM, hematologic malignancy; ICU, intensive care unit; IVIG, intravenous immunoglobulin; LRD, lower respiratory tract disease; SOT, solid organ transplants; URI, upper respiratory tract infection.

### Clinical Factors Related to HMPV Pneumonia

**Figure 3:**
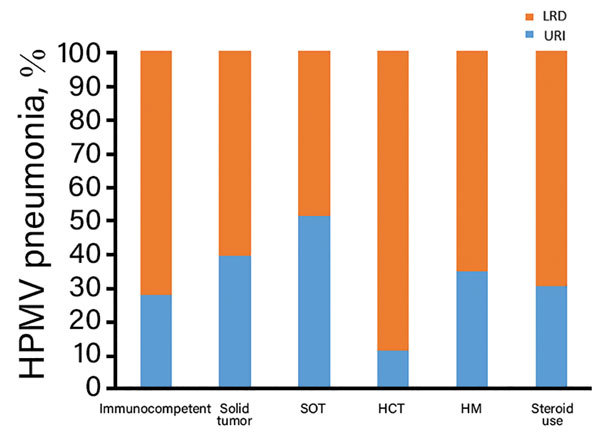
Proportion of HMPV-infected patients with various underlying diseases having HMPV pneumonia, South Korea. HCT, hematopoietic stem cell transplantation; HM, hematologic malignancy; HMPV, human metapneumovirus; LRD, lower respiratory tract disease; SOT, solid organ transplants; URI, upper respiratory infection.

We compared clinical characteristics for patients with HMPV and URI or pneumonia by using logistic regression analysis to identify factors associated with HMPV pneumonia (Table 3). Univariate analysis showed that median age was higher in patients with pneumonia than in those with URI (66.0 vs. 59.0 years, odds ratio [OR] 1.02, 95% CI 1.01–1.03; p<0.001). Current cigarette smoking (OR 2.10, 95% CI 1.47–2.99; p<0.001) and diabetes (OR 1.47, 95% CI 1.08–2.01; p = 0.02) were also significantly associated with HMPV pneumonia. The proportion of HMPV pneumonia was higher for immunocompetent patients (73%, 333/459) than for patients with solid tumors (61%, 106/174) or SOT (49%, 29/59) (p<0.001), However, differences for patients with HCT (89%, 8/9), HM (66%, 38/58), and steroid use (70%, 63/90) were not statistically significant (p = 0.36) (Figure 3).

**Table 3:** Clinical characteristics of patients associated with human metapneumovirus pneumonia, South Korea*

Characteristic	URI, n = 270	LRD, n = 579	Univariate analysis			Multivariable analysis	
			OR (95% CI)	p value		OR (95% CI)	p value
Age	59.0 (49.3–69.8)	66.0 (56.0–74.0)	1.02 (1.01–1.03)	<0.001		1.02 (1.01–1.03)	0.001
Male sex	121 (45)	321 (55)	1.57 (1.17–2.09)	0.002		0.95 (0.65–1.40)	0.81
Smoking				<0.001			0.01
Never	209 (77)	356 (61)	1			1	
Former	12 (4)	39 (7)	1.91 (0.98–3.73)	0.59		1.66 (0.80–3.43)	0.17
Current	51 (19)	182 (31)	2.10 (1.47–2.99)	<0.001		2.07 1.30–3.29)	0.002
Body mass index, kg/m^2^	23.4 (21.0–25.7)	22.7 (20.1–25.4)	0.97 (0.94–1.00)	0.09		0.97 (0.93–1.00)	0.07
Diabetes mellitus	79 (29)	217 (37)	1.47 (1.08–2.01)	0.02		1.37 (0.96–1.95)	0.08
Community-acquired pneumonia	238 (88)	516 (89)	0.83 (0.53–1.30)	0.41		1.10 (0.68–1.79)	0.69
Other pathogen	63 (23)	140 (24)	0.92 (0.66–1.30)	0.64		1.05 (0.73–1.52)	0.80
Underlying condition				0.002			0.003
Immunocompetent	126 (47)	333 (58)	1			1	
Solid tumor	68 (25)	106 (18)	0.59 (0.41–0.85)	0.005		0.57 (0.38–0.84)	0.005
SOT	30 (11)	29 (5)	0.37 (0.21–0.63)	<0.001		0.35 (0.19–0.65)	0.001
HCT	1 (0.4)	8 (1)	3.03 (0.38–24.45)	0.30		3.75 (0.44–31.85)	0.23
HM	20 (7)	38 (7)	0.72 (0.40–1.28)	0.26		0.79 (0.43–1.45)	0.45
Steroid use	27 (10)	63 (11)	0.88 (0.54–1.45)	0.62		0.72 (0.43–1.22)	0.22

*Values are no. (%) or median (interquartile range). HT, hematologic stem cell transplantation; HM, hematologic malignancy; LRD, lower respiratory tract disease; OR, odds ratio; SOT, solid organ transplants; URI, upper respiratory tract infection.

Multivariable logistic regression analysis showed that age (OR 1.02, 95% CI 1.01–1.03; p = 0.001) and current smoking (OR 2.07, 95% CI 1.30–3.29; p = 0.002) were independently associated with HMPV pneumonia, and that diabetes showed marginal significance (OR 1.37, 95% CI 0.96–1.95; p = 0.08). The proportion of HMPV pneumonia was significantly lower for patients with solid tumors (OR 0.57, 95% CI 0.38–0.84; p = 0.005) or SOT (OR 0.35, 95% CI 0.19–0.65; p = 0.001) than for immunocompetent patients; no major differences were observed for the HCT, HM, and steroid use groups.

### Clinical Factors Related to 30-Day Mortality Rates in HMPV-Infected Patients

We found that current smoking (OR 1.92, 95% CI 1.04–3.56; p = 0.04), BMI (OR 0.89, 95% CI 0.79–0.95; p = 0.001), and underlying immunocompromised status such as patients with solid tumors (OR 3.00, 95% CI 1.48–6.08; p = 0.002), but not SOT, were significantly associated with 30-day mortality rates in HMPV-infected patients (Table 4). The association of 30-day mortality rate was highest for HCT patients (OR 7.91, 95% CI 1.52 – 41.1; p<0.01). Multivariable logistic regression analysis showed that BMI (OR 0.86, 95% CI 0.79–0.95; p = 0.002) and underlying immunocompromised status such as patients with solid tumor (OR 3.43, 95% CI 1.58–7.44; p = 0.002) remained associated factors with 30-day mortality rate.

**Table 4:** Clinical characteristics associated with 30-day mortality rate for patients infected with HMPV, South Korea*

Characteristic	Recovered, n = 802	30-day mortality rate, n = 47	Univariate analysis			Multivariable analysis	
			OR (95% CI)	p value		OR (95% CI)	p value
Age	63.5 (53.0–73.0)	67.0 (55.0–77.0)	(0.99–1.04)	0.15		1.02 (1.00–1.05)	0.11
Male sex	416 (52)	26 (55)	0.87 (0.48–1.57)	0.65			
Smoking				0.04			0.54
Never	540 (67)	25 (53)	1			1	
Former	48 (6)	3 (6)	1.35 (0.39–4.63)	0.63		0.67 (0.15–3.04)	0.61
Current	214 (27)	19 (40)	1.92 (1.04–3.56)	0.04		1.35 (0.69–2.64)	0.39
Body mass index, kg/m^2^	23.1 (20.5–25.6)	20.5 (18.2–24.1)	0.89 (0.79–0.95)	0.001		0.86 (0.79–0.95)	0.002
Diabetes mellitus	277 (35)	19 (40)	1.29 (0.71–2.35)	0.41		1.48 (0.77–0.95)	0.24
Community-acquired pneumonia	715 (89)	40 (85)	0.70 (0.30–1.60)	0.39		0.83 (0.32–2.11)	0.69
Other pathogen	193 (24)	10 (21)	0.86 (0.42–1.76)	0.68		0.75 (0.35–1.60)	0.45
Underlying condition				0.02			0.02
Immunocompetent	443 (55)	16 (34)	1				
Solid tumor	157 (20)	17 (36)	3.00 (1.48–6.08)	0.002		3.43 (1.58–7.44)	0.002
SOT	59 (7)	0	0.00 (0.00–0.00)	1.00		0.00 (0.00–0.00)	1.00
HCT	7 (0.9)	2 (4)	7.91 (1.52–41.1)	0.01		9.19 (1.47–57.37)	0.02
HM	53 (7)	5 (11)	2.61 (0.92–7.42)	0.07		3.44 (1.14–10.34)	0.03
Steroid use	83 (10)	7 (15)	2.34 (0.93–5.85)	0.07		2.40 (0.91–6.34)	0.08

*Values are no. (%) or median (interquartile range). HCT, hematologic stem cell transplantation; HM, hematologic malignancy; OR, odds ratio; SOT, solid organ transplants.

### Radiologic Findings for HMPV Pneumonia

**Figure 4:**
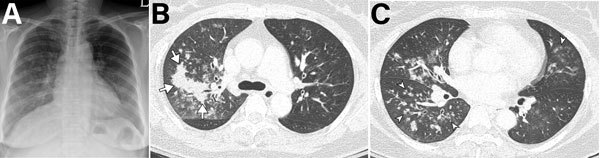
Imaging of 55-year-old immunocompetent woman with human metapneumovirus pneumonia, South Korea. A) Initial chest radiograph showing ill-defined patchy and nodular ground-glass opacities in the right lung and left lower lung zone. B, C) Chest computed tomography showing irregular nodular consolidation (arrows in panel B) and multiple ill-defined centrilobular nodular opacities (arrowheads in panel C) with mild bronchial wall thickening. Five days later, the lesions had resolved completely.

**Figure 5:**
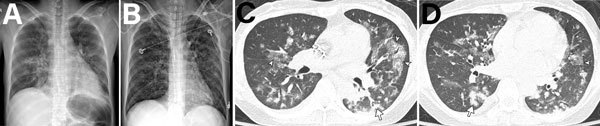
Imaging of 38-year-old woman in South Korea who underwent liver transplantation 2 months earlier, followed by immunosuppressant therapy, who visited the emergency department because of dyspnea, chills, and fever. A) Initial chest radiograph showing bilateral peribronchial infiltration in the central areas of both lungs and minimal pleural effusion. B) Five days later, the patient was admitted to the intensive care unit and underwent intubation because of progressive dyspnea. Radiograph showed extensive bilateral peribronchial nodular infiltrates. C, D) Computed tomography performed on the same day as in panel B showing multifocal peribronchial nodular consolidation (arrows), ground-glass opacities (arrowheads), and irregular small nodules, along with small amounts of bilateral pleural effusion. The patient was given ribavirin and prophylactic antimicrobial drugs and recovered after 10 days in the intensive care unit.

We detected HMPV pneumonia radiographically for 68% (579/849) of all patients, and 56.0% (324/579) of these patients had bilateral involvement. Median number of involved zones in patients with HMPV pneumonia was 3 (range 2–5). To evaluate CT findings for HMPV pneumonia, we analyzed 251 patients without another pathogen who had CT performed at the time of diagnosis (Table 5). Of these patients, 76% (192/251) showed bilateral involvement. Of the patients with HMPV pneumonia, 69% (174/251) had centrilobular nodules, 43% (107/251) had consolidations, and 79% (199/251) had ground-glass opacities (Figure 4). Macronodules (45%, 114/251) and bronchial wall thickening (88%, 222/251) were common findings, whereas mediastinal lymphadenopathy (27%, 68/251) and pleural effusion (22%, 56/251) were less common. Intraclass correlation coefficients of radiologists for CT findings (percent extents of centrilobular nodules, consolidation, and ground-glass opacities) ranged from 0.84 to 0.95 (p<0.001). The number of involved lobes was greater and pleural effusion was more frequent in immunocompromised patients than in immunocompetent patients (Figure 5; Appendix Table 3).

**Table 5:** CT findings for 251 patients with HMPV pneumonia without another pathogen, South Korea*

Characteristic	Immunocompetent	Solid tumors	SOT	HCT	HM	Steroid use	Total
Patients with HMPV pneumonia without another pathogen	259	81	36	7	29	41	453
Patients who underwent CT scans	138	43	26	3	23	18	251
Bilateral	100 (72.5)	34 (79.1)	21 (80.8)	3 (100)	20 (87.0)	14 (77.8)	192 (76)
No. involved lobes	3 (2–6)	5 (3–6)	4 (2–5)	6 (6–6)	5 (3–6)	4 (3–5)	4 (2–6)
Macronodule	67 (49)	19 (44)	11 (31)	1 (33)	12 (52)	4 (22)	114 (45)
Patients with centrilobular nodules	101 (73)	28 (65)	17 (47)	2 (67)	13 (57)	13 (72)	174 (69)
Extent of centrilobular nodules	10 (5–30)	10 (0–20)	10 (0–20)	20 (10–20)	10 (0–20)	10 (0–23)	10 (5–20)
Patients with consolidation	57 (41)	20 (47)	13 (50)	0	8 (35)	9 (50)	107 (43)
Extent of consolidation	0 (0–10)	0 (0–15)	3 (0–10)	0 (0–0)	0 (0–10)	8 (0–20)	0 (0–10)
Patients with ground-glass opacity	109 (79)	38 (88)	21 (81)	2 (67)	15 (65)	14 (78)	199 (79)
Extent of ground-glass opacity	10 (10–20)	10 (10–30)	10 (10–23)	10 (10–10)	10 (5–20)	18 (9–33)	10 (10–20)
Bronchial wall thickening	121 (88)	35 (81)	25 (96)	3 (100)	1 (4)	17 (94)	222 (88)
Bronchiectasis	17 (12)	4 (9)	4 (15)	1 (33)	3 (13)	5 (28)	34 (14)
Cavitation	1 (0.7)	0	0	0	0	1 (6)	2 (1)
Lymphadenopathy	40 (29)	14 (33)	6 (23)	0 (0)	2 (9)	6 (33)	68 (27)
Pleural effusion	24 (17)	15 (35)	7 (27)	1 (33)	5 (22)	4 (22)	56 (22)

*Values are no. (%) or median (interquartile range) unless otherwise indicated. CT, computed tomography; HCT, hematologic stem cell transplantation; HM, hematologic malignancy; LRD, lower respiratory tract disease; SOT, solid organ transplants; URI, upper respiratory tract infection.

## Discussion

We found that HMPV pneumonia in adults was associated with older age, current cigarette smoking, and underlying diseases. Lower BMI and immunocompromised state, except SOT, were associated with 30-day mortality rates in HMPV-infected patients. Chest radiographs showed bilateral lung involvement in 56% of patients, and CT scans showed bilateral lung involvement in 76% of patients. Bronchial wall thickening, ground-glass opacities, and ill-defined centrilobular nodules were common CT findings in patients with HMPV pneumonia.

Recent studies have demonstrated that low BMI was a major risk factor for hospitalization of patients with pneumonia (*23*,*24*). A U-shaped relationship between BMI and pneumonia has been shown in a recent meta-analysis, in which an underweight condition (BMI <18.5) and morbid obesity (BMI >40) are both associated with the risk for community-acquired pneumonia (*25*). Conversely, being overweight and obese (BMI 25.0–39.9) were major factors in reducing the risk for death from pneumonia (*26*). In our study, the risk for HMPV pneumonia associated with BMI was not evident because only 14 patients were underweight, and there was no patient with a BMI >32. However, lower BMI increased the mortality rate for HMPV pneumonia. This finding coincides with the obesity survival paradox, which shows a decreased pneumonia mortality rate for overweight and obese patients (*26*).

Although most young healthy adults with HMPV infection are asymptomatic or have mild symptoms, HMPV pneumonia can cause severe symptoms in elderly (*7*) and immunocompromised (*14*,*17*,*27*,*28*) patients. Mortality rates for HMPV pneumonia have been reported to be higher (up to 40%) in HCT recipients (*15*). A recent meta-analysis showed that the overall HMPV mortality rate for HCT recipients was 6% and that the HMPV-associated mortality rate was 5.9 times higher in patients with pneumonia (27%) than in patients with URI (*16*). In our study, although the number of HCT patients was small (n = 9), the mortality rate was highest for HCT recipients, and the all-cause 30-day mortality rate was 22%. Conversely, the proportion of SOT patients with pneumonia was lower than that for immunocompetent patients, and mortality rates were lower for SOT patients than for all other groups.

The proportion of patients with HMPV pneumonia among those with HMPV infection was smaller for the solid tumor and SOT groups than for immunocompetent patients, whereas the mortality rate was highest for HCT recipients, followed by those with HM. This finding might be explained by the fact that immunocompetent patients visited the tertiary hospital only when respiratory symptoms were not eliminated by management in a primary clinic, whereas patients about to receive organ transplants or HCT visited the tertiary hospital if they had less severe symptoms, and had more periodical follow-up visits. Therefore, the proportion of pneumonia in the immunocompetent group was high in this study.

In our study, use of ribavirin was more common in ICU admitted patients, and mortality rates were also higher in patients who received ribavirin. However, this study was not designed to analyze the efficacy of ribavirin, and ribavirin might have been used more often for treatment in severely ill patients. Thus, the effect of ribavirin use was not determined in this study.

We found that, radiologically, most patients with HMPV pneumonia had bilateral involvement; the main findings on CT images included ill-defined centrilobular nodules, ground-glass opacities, and irregular nodular consolidations. These findings suggested bronchitis and bronchiolitis and were consistent with those of previous studies (*28*–*32*). Findings of bronchial wall thickening, bronchiolitis, and centrilobular nodules were likely caused by pathogenesis of HMPV pneumonia, which affects the airways and lung epithelia and induces inflammatory cascades (*33*–*35*). Because these CT findings are also observed for bacterial pneumonia, they might be insufficient for excluding the possibility of another pathogen. CT scans of patients infected with HMPV but without another pathogen showed that the number of involved lobes and the frequency of pleural effusion were greater in the immunocompromised patients than in the immunocompetent patients.

This study had several limitations. First, patients with mild symptoms and no definite lesions by chest radiography might not undergo CT examinations. Therefore, patients with mild pneumonia that is not clearly visible on chest radiographs and who have not undergone CT scans might be classified as being in the URI group. Second, patients with low virus loads might be undetected because the Seeplex PCR has a sensitivity of 88% and the Anyplex test has a sensitivity of 96% for detecting HMPV infection (*36*). In addition, we did not routinely assess virus loads in specimens by using quantitative methods. Therefore, the effect of virus load on development of HMPV pneumonia could not be assessed, although virus load was shown not to be a major factor for HCT recipients (*14*). Third, we routinely checked patients who had respiratory infection symptoms by using nasopharyngeal swab specimens or blood cultures in our hospital. However, the decision for testing was made by clinicians, and patients with subtle respiratory symptoms might not be identified.

In conclusion, we report clinical and radiologic findings for HMPV infection in patients with various immune states. About half of these patients, even those who were immunocompetent, had HMPV pneumonia. Older age and current smoking were strongly associated with HMPV pneumonia, and the mortality rate was high in HCT recipients. CT showed that bilateral bronchial wall thickening, ground-glass opacities, and ill-defined centrilobular nodules were common.

Appendix Additional information on clinical and radiologic characteristics of human metapneumovirus infections in adults, South Korea.
