# Correction: Vol. 25, No. 1

**DOI:** 10.3201/eid2502.C32502

**Published:** 2019-02

**Keywords:** errata, erratum, correction

Author Sang-Ho Choi’s name was listed incorrectly and author affiliations were unclear in Clinical and Radiologic Characteristics of Human Metapneumovirus Infections in Adults, South Korea (H.J. Koo et al.). The article has been corrected online (https://wwwnc.cdc.gov/eid/article/25/1/18-1131_article).

