# Supplementary material for: Clinical and Radiologic Characteristics of Human Metapneumovirus Infections in Adults, South Korea
Source: Emerg Infect Dis. 2019 Jan;25(1):15–24. doi: 10.3201/eid2501.181131 (PMC6302610; doi:10.3201/eid2501.181131)
Supplement: Appendix — Additional information on clinical and radiologic characteristics of human metapneumovirus infections in adults, South Korea. [file 18-1131-Techapp-s1.pdf]

Article DOI: https://doi.org/10.3201/eid2501.181131

# Clinical and Radiologic Characteristics of Human Metapneumovirus Infections in Adults, South Korea

## Appendix

**Appendix Table 1.** Underlying diseases in patients infected with HMPV, South Korea*

| Disease or condition | | Subtype | | |
|---|---|---|---|---|
| Solid tumors (174) | SOT (59) | HCT (9) | of HM (58) | Steroid use (90) |
| Lung cancer (50) | Kidney (37) | MSD (4) | Multiple myeloma (19) | Chronic obstructive pulmonary disease (24) |
| Breast cancer (33) | Liver (16) | MUD (1) | Acute myeloid leukemia (17) | Interstitial lung disease (18) |
| Hepatocellular carcinoma (15) | Pancreas and kidney (2) | Haploidentical (3) | Diffuse large B cell lymphoma (8) | Asthma (12) |
| Stomach cancer (15) | Heart (4) | Autologous (1) | Acute lymphoblastic leukemia (4) | Pontine infarction or intracranial hemorrhage (7) |
| Colorectal cancer (12) | | | Chronic myeloid leukemia (2) | Systemic lupus erythematosus (4) |
| Esophageal cancer (6) | | | Myelodysplastic syndrome (2) | Ulcerative colitis (3) |
| Prostate cancer (6) | | | Red cell aplasia (1) | Demyelinating polyneuropathy (3) |
| Brain tumor (5) | | | Mantle cell lymphoma (1) | Adrenal insufficiency (2) |
| Endometrial cancer (4) | | | B cell lymphoblastic lymphoma (1) | Rheumatoid arthritis (2) |
| Head and neck cancer (4) | | | Primary CNS lymphoma (1) | Idiopathic vasculitis (2) |
| Tubo-ovarian cancer (3) | | | Angioblastic T-cell lymphoma (1) | Behcet disease (1) |
| Bile duct cancer (3) | | | Acute leukemia of unspecified cell type (1) | Myasthenia gravis (1) |
| Pancreas cancer (3) | | | | Takayasu arteritis (1) |
| GIST (3) | | | | CNS vasculitis (1) |
| Thymic cancer (2) | | | | Autoimmune hepatitis (1) |
| Bladder cancer (2) | | | | Perineuritis (1) |
| Adrenal cortical carcinoma (2) | | | | Cushing disease (1) |
| Lung cancer and stomach cancer (1) | | | | Adult-onset Still’s disease (1) |
| Cholangiocarcinoma (1) | | | | Idiopathic thrombocytopenic purpura (1) |
| Renal cell carcinoma (1) | | | | ANCA associated vasculitis (1) |
| Malignant fibrous histiocytoma (1) | | | | Critical illness polyneuropathy (1) |
| Papillary thyroid carcinoma (1) | | | | Polymyalgia rheumatica (1) |
| Cancer of unknown origin (1) | | | | Sarcoidosis (1) |

*Values in parentheses are no. patients. Empty spaces indicate fewer diseases. CNS, central nervous system; GIST, gastrointestinal stromal tumor; HCT, hematologic stem cell transplantation; HM, hematologic malignancy; HMPV, human metapneumovirus; MSD, matched sibling donor; MUD, matched unrelated donor; SOT, solid organ transplants.

**Appendix Table 2.** Other respiratory pathogens identified in patients infected with HMPV, South Korea*

| Pathogen | Immunocompetent, n = 459 | Solid tumors, n = 174 | Type of SOT, n = 59 | Type of HCT, n = 9 | Type of HM, n = 58 | Steroid use, n = 90 |
|---|---|---|---|---|---|---|
| Bacteria | 48 | 20 | 16 | 1 | 11 | 20 |
| Virus | 32 | 14 | 7 | 0 | 6 | 5 |
| Fungi | 3 | 5 | 0 | 1 | 0 | 0 |
| Bacteria and virus | 6 | 1 | 0 | 0 | 2 | 3 |
| Bacteria and fungi | 0 | 1 | 0 | 0 | 0 | 0 |
| Virus and fungi | 0 | 0 | 0 | 0 | 0 | 1 |
| | 11 *Streptococcus pneumoniae* | 6 *Klebsiella pneumoniae* | 5 *S. pneumonia* | 1 MSSA | 4 MSSA | 5 *K. pneumoniae* |
| | 6 *K. pneumoniae* | 5 MSSA | 4 G+ cocci (unknown) | 1 *Aspergillus* spp. | 2 *Acinetobacter baumannii* | 3 *S. pneumoniae* |

| Pathogen | Immunocompetent, n = 459 | Solid tumors, n = 174 | Type of SOT, n = 59 | Type of HCT, n = 9 | Type of HM, n = 58 | Steroid use, n = 90 |
|---|---|---|---|---|---|---|
| | 5 *P. aeruginosa* | 4 *S. pneumoniae* | 1 *Pseudomonas aeruginosa*, CRAB | | 1 MRSA | 3 *P. aeruginosa* |
| | 5 MRSA | 1 MRSA | 1 CRPA | | 1 *K. pneumoniae* | 3 *A. baumannii* |
| | 4 MSSA | 1 *Haemophilus influenzae* | 1 *K. pneumonia* | | 1 *P. aeruginosa* | 2 MRSA |
| | 4 *A. baumannii* | 1 *Moraxella catarrhalis* | 1 *K. pneumoniae, Aspergillus* spp. | | 1 MSSA, S. *pneumoniae* | 1 MSSA |
| | 4 *Haemophilus influenza* | 1 *K. pneumoniae, S. pneumoniae* | 1 *Escherichia coli* | | 1 *K. pneumoniae, S. pneumoniae* | 1 *Acinetobacter lwoffii* |
| | 1 *Klebsiella oxytoca* | 1 *K. oxytoca, P. aeruginosa* | 1 *Mycoplasma pneumonia* | | 3 Rhinovirus | 1 *A. baumannii,* MRSA |
| | 1 MRSA, CRAB | 6 Rhinovirus | 1 MSSA | | 1 PIV-4 | 1 *K. pneumoniae* |
| | 1 MRSA, *Enterobacter cloacae* | 3 Coronavirus OC43 | 1 Influenza virus B | | 1 PIV-3 | 1 Coronavirus OC43 |
| | 1 MRSA, *P. aeruginosa* | 1 Influenza virus A | 1 RSV-A | | 1 RSV-A | 1 Influenza virus B |
| | 1 MRSA, CRAB | 1 Enterovirus | 1 Cytomegalovirus | | 1 MSSA, PIV-1 | 1 Rhinovirus |
| | 1 *S. pneumoniae, P. aeruginosa* | 1 Coronavirus 229E | 1 Coronavirus NL63 | | 1 MSSA, rhinovirus | 1 Influenza virus A |
| | 1 *A. baumannii, S. pneumoniae* | 1 PIV-3 | 2 Rhinovirus | | | 1 RSV-B |
| | 1 *Haemophilus influenzae, K. pneumoniae, S. pneumoniae* | 1 Coronavirus OC43 | 1 Adenovirus | | | 1 *M. catarrhalis,* PIV-4, influenza virus A |
| | 1 *Corynebacterium striatum, A. baumannii* | 4 *Aspergillus* spp. | | | | 1 *P. aeruginosa,* PIV-3 |
| | 14 Rhinovirus | 1 *Rhizopus* spp. | | | | 1 MRSA, influenza virus B |
| | 5 Influenza virus A | 1 *K. pneumoniae,* PIV-3 | | | | 1 Coronavirus OC43, *Aspergillus* spp. |
| | 5 Coronavirus OC43 | 1 MSSA, *Aspergillus* spp. | | | | |
| | 2 PIV-3 | | | | | |
| | 2 Adenovirus | | | | | |
| | 1 Coronavirus 229E | | | | | |
| | 1 RSV-A | | | | | |
| | 1 Enterovirus | | | | | |
| | 1 Rhinovirus, RSV-A | | | | | |
| | 2 *Aspergillus* spp. | | | | | |
| | 1 *Rhizopus* spp. | | | | | |
| | 1 *S. pneumoniae*, rhinovirus | | | | | |
| | 1 *S. pneumoniae*, coronavirus OC43 | | | | | |
| | 1 *S. pneumoniae,* influenza virus A | | | | | |
| | 1 *K. pneumoniae*, PIV-1 | | | | | |
| | 1 MSSA, influenza virus B | | | | | |
| | 1 *P. aeruginosa*, influenza virus A | | | | | |

*Values are no. patients. Empty spaces indicate fewer pathogens. The order from top to bottom of each column is bacteria, virus, fungi, and other pathogen. CRAB, carbapenem-resistant *A. baumannii*; CRPA, carbapenem-resistant *P. aeruginosa*; HCT, hematologic stem cell transplantation; HM, hematologic malignancy; HMPV, human metapneumovirus; PIV, parainfluenza virus; RSV, respiratory syncytial virus; MRSA, methicillin-resistant *Staphylococcus aureus*; MSSA, methicillin-susceptible *S. aureus*; MRSA, methicillin resistant *Staphylococcus aureus*; SOT, solid organ transplants.

**Appendix Table 3.** Extent of HMPV pneumonia by CT for 251 immunocompetent and immunocompromised patients without other pathogens, South Korea*

| Characteristic | Immunocompetent, n = 138 | Immunocompromised, n = 113 | p value |
|---|---|---|---|
| Bilaterality | 99 (72) | 92 (81) | 0.07 |
| No. involved lobes | 3.6 ± 1.9 | 4.3 ± 1.7 | 0.003 |
| Macronodule | 67 (49) | 47 (42) | 0.27 |
| Presence of centrilobular nodules | 108 (78) | 81 (72) | 0.23 |
| Extent of centrilobular nodules, % | 16.0 ± 13.2 | 14.2 ± 13.7 | 0.29 |
| Presence of consolidation | 60 (43) | 53 (47) | 0.59 |
| Extent of consolidation, % | 7.1 ± 10.7 | 7.7 ± 10.8 | 0.68 |
| Presence of ground glass opacity | 116 (84) | 100 (88) | 0.31 |
| Extent of ground-glass opacity | 15.0 ± 12.8 | 18.5 ± 15.9 | 0.06 |
| Bronchial wall thickening | 121 (88) | 101 (89) | 0.68 |
| Bronchiectasis | 17 (12) | 17 (15) | 0.53 |
| Cavitation | 1 (0.7) | 1 (0.9) | 1.00 |
| Lymphadenopathy | 40 (29) | 27 (24) | 0.36 |
| Pleural effusion | 24 (17) | 32 (28) | 0.04 |

*Values are no. (%) or mean ± SD. CT, computed tomography; HMPV, human metapneumovirus.
